# Emulsifying Activity and Stability of a Non-Toxic Bioemulsifier Synthesized by *Microbacterium* sp. MC3B-10

**DOI:** 10.3390/ijms140918959

**Published:** 2013-09-13

**Authors:** Juan Carlos Camacho-Chab, Jean Guézennec, Manuel Jesús Chan-Bacab, Elvira Ríos-Leal, Corinne Sinquin, Raquel Muñiz-Salazar, Susana del C. De la Rosa-García, Manuela Reyes-Estebanez, Benjamín Otto Ortega-Morales

**Affiliations:** 1 Center of Environmental Microbiology and Biotechnology, Autonomous University of Campeche, Colonia Buenavista, San Francisco de Campeche 24039, Campeche, Mexico; E-Mails: juanccam@uacam.mx (J.C.C.-C.); manjchan@uacam.mx (M.J.C.-B.); scdelaro@uacam.mx (S.C.R.-G.); reyes_estebanez@hotmail.com (M.R.-E.); 2 Ifremer Laboratoire Biotechnologies et Molécules Marines, Nantes B. P. 21105, France; E-Mails: guezennec.jean@wanadoo.fr (J.G.); Corinne.Sinquin@ifremer.fr (C.S.); 3 Department of Biotechnology and Bioengineering, Center for Research and Advanced Studies of the National Polytechnic Institute (CINVESTAV), Mexico City 07360, Federal District, Mexico; E-Mail: erios@cinvestav.mx; 4 Laboratory of Molecular Ecology and Epidemiology, School of Health Sciences, Autonomous University of Baja California, Tijuana 22390, Baja California, Mexico; E-Mail: ramusal@uabc.edu.mx

**Keywords:** bioemulsifier, emulsifying stability, non-toxic, *Microbacterium* sp., biotechnology

## Abstract

A previously reported bacterial bioemulsifier, here termed microbactan, was further analyzed to characterize its lipid component, molecular weight, ionic character and toxicity, along with its bioemulsifying potential for hydrophobic substrates at a range of temperatures, salinities and pH values. Analyses showed that microbactan is a high molecular weight (700 kDa), non-ionic molecule. Gas chromatography of the lipid fraction revealed the presence of palmitic, stearic, and oleic acids; thus microbactan may be considered a glycolipoprotein. Microbactan emulsified aromatic hydrocarbons and oils to various extents; the highest emulsification index was recorded against motor oil (96%). The stability of the microbactan-motor oil emulsion model reached its highest level (94%) at 50 °C, pH 10 and 3.5% NaCl content. It was not toxic to *Artemia salina* nauplii. Microbactan is, therefore, a non-toxic and non-ionic bioemulsifier of high molecular weight with affinity for a range of oily substrates. Comparative phylogenetic assessment of the 16S rDNA gene of *Microbacterium* sp. MC3B-10 with genes derived from other marine *Microbacterium* species suggested that this genus is well represented in coastal zones. The chemical nature and stability of the bioemulsifier suggest its potential application in bioremediation of marine environments and in cosmetics.

## 1. Introduction

Microorganisms produce a wide variety of high and low molecular weight biosurfactants. These active molecules include proteins, polysaccharides, lipopeptides, glycolipids, flavolipids, phospholipids and lipopolysaccharides [1]. High molecular weight biosurfactants, usually referred to as bioemulsifiers, can form and stabilize oil-in-water or water-in-oil emulsions, work at low concentrations and exhibit considerable substrate specificity [2]. The chemical diversity and functional properties of these compounds result in a broad spectrum of potential applications in sectors as diverse as agriculture, cosmetics, environmental, food, leather, paper, pharmaceutical and textile industries [2,3]. Biologically derived surface-active molecules have advantages over their synthetic counterparts, including biodegradability, lower toxicity, novel structural chemistry and high stability at extreme temperature, salinity and pH [2,4]. Some bioemulsifiers, e.g., emulsan [5], have been extensively characterized and reached commercial applications, chiefly in the bioremediation sector.

Bioprospection of various habitats, in particular marine environments [6,7], has yielded many novel biomolecules produced by microorganisms. An earlier report showed that the extracellular biopolymer produced by *Microbacterium* sp. MC3B-10 emulsified hexane, decane and hexadecane at higher efficiencies than commercial surfactants Triton^®^ X-100 and Tween^®^ 80 [8]. This extracellular biopolymer will be referred to as microbactan after its bacterial producer. The goal of the present study was to characterize in greater detail this extracellular biopolymer and determine the emulsifying stability as a function of key variables.

## 2. Results and Discussion

### 2.1. Characterization of Microbactan

Microbactan was produced by fermentation in shaker flasks; no attempt was made at this time to optimize its production using a bioreactor, where more strict control of variables can be achieved. The reproducibility of production conditions employed in this study was confirmed by the yield and the primary chemical profile of the biopolymer, which was comparable to the previous report [8]. Our previous work showed that microbactan was dominated by carbohydrates and proteins, and was preliminarily considered a glycoprotein. The present study also revealed the presence of lipids, shown by the peak at 2927 cm^−1^ in the FT-IR analysis, which represents the asymmetric stretch (C–H) of –CH_2_ groups combined with that of –CH_3_ groups in lipids [9]. Quantitative assessment by absorbance showed that the lipid signature detected by FT-IR corresponded to a lipid content of 8% ± 0.5%. The gas chromatograph analysis of the lipid fraction revealed the presence of palmitic (C-16), stearic (C-18) and oleic (C-18:1) acids. Other fatty acids, found at lower levels, were myristoleic (C-14:1) and linoleic (C-18:2). The lipid content and the previously reported chemical composition of ~90% carbohydrates and proteins [8] represented almost 100% of the total mass of the microbactan on a dry weight basis, suggesting that this biopolymer is a class of glycolipoprotein. The fatty acids detected (palmitic, stearic and oleic) have been reported in bioemulsifiers produced by *Yarrowia lipolytica* [10] and *Penicillium* sp. [11]. However, the presence of other substituents of extracellular biopolymers such as pyruvil, succinyl and sulfates, which often occur at minor levels [4,9], was not investigated.

High performance size exclusion chromatography showed that microbactan had a molecular weight (MW) approaching 700 kDa and a polydispersity index (Mw/number average molecular weight) of 1.3; a value close to 1 denotes a polymer with homogeneous monomer grouping. These results indicate that microbactan is a homogeneous high molecular weight glycolipoprotein, a finding that is consistent with previous reports showing that, in general, bioemulsifiers are high molecular weight polymers [1,12]. The commercial bioemulsifier alasan^®^ has a molecular weight of approximately 1000 kDa [13]. Most bacterial bioemulsifiers are polymers of either carbohydrates or proteins, or even glycoproteins [1,6]. Thavasi and colleagues report marine-derived glycolipoprotein emulsifiers from *Corynebacterium kutcheri* and *Bacillus megaterium* [14,15]. The putative glycolipoprotein nature of microbactan is, therefore, to some extent unusual among marine bioemulsifiers.

Microbactan was shown by the modified double diffusion test to be a non-ionic emulsifier. The ionic character of polymers is one of the features that contribute strongly to the functional properties of emulsifiers; this property is often reported for synthetic emulsifiers or novel emulsifying formulations used for a range of applications. Despite the importance of this feature, it is rarely reported in newly discovered bioemulsifiers. The non-ionic nature of emulsifiers greatly contributes to emulsion stability, generating a number of short-range repulsive forces, such as steric, hydration, and thermal fluctuation interactions, which prevent the droplets from getting too close together [16].

### 2.2. Emulsifying Potential

Tables 1 and 2 show the emulsifying activity of microbactan and control commercial emulsifiers tested against different substrates at different times. Statistical analysis showed significant differences between microbactan and these controls (*F* = 84.24; *p* < 0.05). Synthetic surfactants Triton^®^ X-100 and Tween^®^ 80 were more efficient than microbactan and the commercial biopolymers, reaching emulsification efficiencies of 100% against oils (with the exception of motor oil), irrespective of time of incubation (24 and 96 h). Microbactan showed stronger emulsifying activities than gum arabic but was comparable to the emulsifying efficiency of xanthan gum, another bacterially-produced biopolymer. The emulsifying activity of microbactan was dependent on the type of substrate (*F* = 16.2; *p* < 0.05); this is consistent with other reports on substrate-specific hydrocarbon metabolism by marine bacteria [17,18]. Microbactan emulsions with the tested substrates were rather stable over time (Tables 1 and 2), varying only slightly between 24 and 96 h.

An established criterion for emulsion-stabilizing capacity is the ability of an emulsifier to maintain at least 50% of the original volume of the emulsion for 24 h [4,19]. Microbactan emulsions remained stable for several months, showing no sign of droplet coalescence after standing at room temperature (28 °C). This extended stability has previously been observed for glycoprotein bioemulsifiers produced by a marine *Antarctobacter* [20]. Emulsifying and surfactant activities are together responsible for important functional properties of bacterial exopolymers. Our analyses revealed that microbactan has emulsifying but not surfactant activity. Surface-active biomolecules are classified as surfactants when they lower the interfacial or surface tension and emulsifiers when they form stable emulsions [19,21]. Overall, our results indicate that microbactan is a true bioemulsifier.

### 2.3. Effect of Temperature, pH and NaCl on Emulsifying Activity

**Figure 1:**
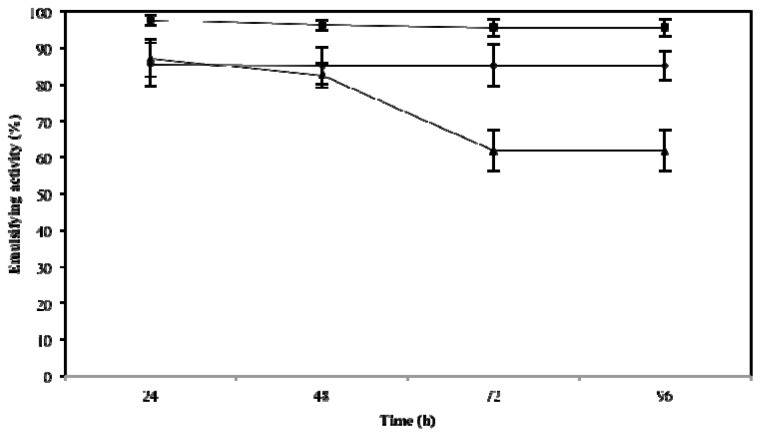
Emulsifying activity of microbactan on motor oil at different temperatures up to 96 h. 5 °C (●), 50 °C (■) and 100 °C (▲). Values represent means ± SD (*n* = 3).

**Figure 2:**
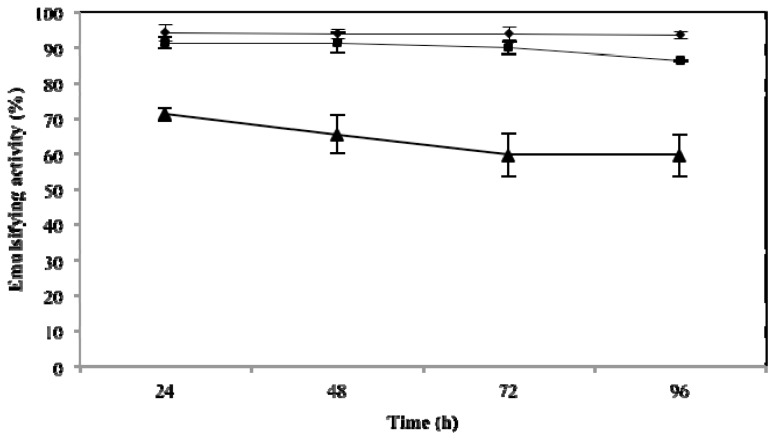
Emulsifying activity of microemulsan on motor oil at various salinities up to 96 h. 3.5% (●), 5% (■) and 10% (▲) NaCl concentration. Values represent means ± SD (*n* = 3).

**Figure 3:**
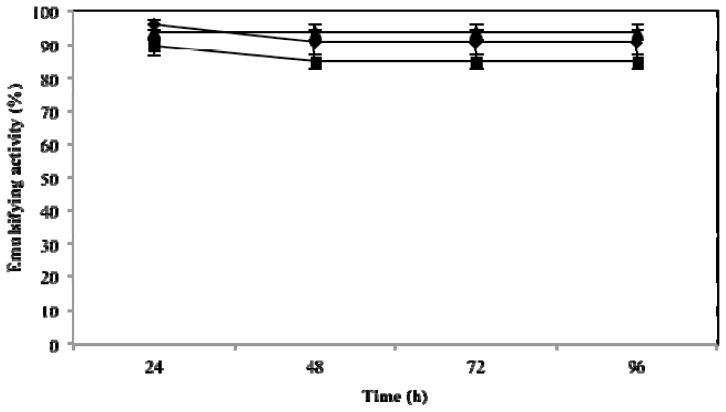
Emulsifying activity of microbactan on motor oil at different pH values. pH 4 (●), pH 7 (■) and pH 10 (▲).Values represent means ± SD (*n* = 3).

The effect of these three key variables that influence emulsifying activity was assessed at 24, 48, 72 and 96 h. As expected, they influenced the emulsifying activity of microbactan, but their effects occurred at different levels. A time-dependent decrease of emulsifying activity was observed at 100 °C (*F* = 38.13; *p* < 0.005). This was not the case for activities at 50 °C and 5 °C. The highest level of activity was found at 50 °C (emulsifying activity of 95.7% ± 2.5%) irrespective of time of incubation (Figure 1). The loss of emulsifying activity at 100 °C can be explained by denaturation of the protein fraction of microbactan during heating [22], as seen with other microbial biosurfactants [23]. Similarly, the activity associated with the upper limit of the range of salinities tested (3.5%, 5%, and 10%) declined as a function of time to level off at 72 and 96 h. The highest levels of activity were observed at 3.5% (Figure 2), not dissimilar to the biosurfactant produced by *Aeromonas* spp., which maintained emulsifying activity up to 5% NaCl [24]. On the other hand, no significant effect on activity was observed as a function of time with the pH values tested (Figure 3). However, slightly higher levels of emulsifying activity were recorded at acid and alkaline pH values, suggesting the ionization of functional groups that resulted in the activation of less surface-active species within the bioemulsifier matrix [25]. In comparison, biodispersan from *Acinetobacter calcoaceticus* A2 had an optimum functional pH value in the range of 9 to 12 for limestone-dispersing activity [26]. It has been shown that the emulsifying activity of certain polymers is modified at different extents when temperature and pH covariate, this is probably due to the synergistic influence of these factors on surface-active proteins, whose conformation and functional groups are influenced as a function of these factors.

These results suggest that microbactan could find application in environmental marine processes such as enhanced oil recovery, cleaning of oil reservoirs and enhancement of biodegradation rates of spilled oils [2]. Bioemulsifiers have a wide diversity of composition and structure and are characterized by improved functionality and stability. Their potential applications include: the oil and petroleum industries, water and soil bioremediation, metal treatment and processing, detergents and laundry supplies, agriculture, textile manufacturing, pulp and paper processing, paints, cosmetics, pharmaceuticals, personal care products and food processing [4,6]. Microbactan has higher emulsifying activity at 50 °C and 3.5% NaCl, conditions typical of intertidal environments [8]. If the molecule remains stable under these conditions for long periods, it may prove specifically useful for bioremediation of polluted intertidal habitats [27]. The stability of the emulsions under diverse conditions, such as temperature, pressure, pH and ionic strength, makes this biopolymer a versatile emulsifier for use in many food and pharmaceutical formulations.

### 2.4. Toxicity of Microbactan

Based solely on the *Artemia salina* toxicity test, microbactan proved innocuous, as expected for a biologically derived surface-active agent; this class of compound is generally biodegradable and non-toxic [2]. Although synthetic surfactants exhibited the highest emulsifying activity in this study, Triton^®^ X-100 (also a non-ionic surface-active), at least, proved to be toxic in our *Artemia* bioassay (Table 3). Polyoxyethylene octyl phenols (the Triton X series) are known to be highly cytotoxic, solubilizing the membrane lipid bilayer [28]. In fact, these synthetic surfactants can actually inhibit aromatic hydrocarbon biodegradation via toxic interactions, making them less suitable for bioremediation purposes [27,29]. The *Artemia salina* toxicity bioassay is a reliable primary screen, given the sensitivity of this crustacean to a wide range of biologically active compounds of diverse chemistries, including pesticide residues, mycotoxins, stream pollutants, anaesthetics, dinoflagellate toxins, morphine-like compounds and oil dispersants [30]. In addition, this bioassay has demonstrated good correlation with other cell-based tests such as tumor cell lines (e.g., KB, P-388, 388, L5178Y and L1210) and mammalian systems [31,32]. Reinforcing this finding, an experimental study on marine biofilm colonization on surfaces coated with microbactan showed that this biopolymer did not affect biofilm formation (unpublished results).

### 2.5. Phylogenetic Reassessment of *Microbacterium* sp. MC3B-10

**Figure 4:**
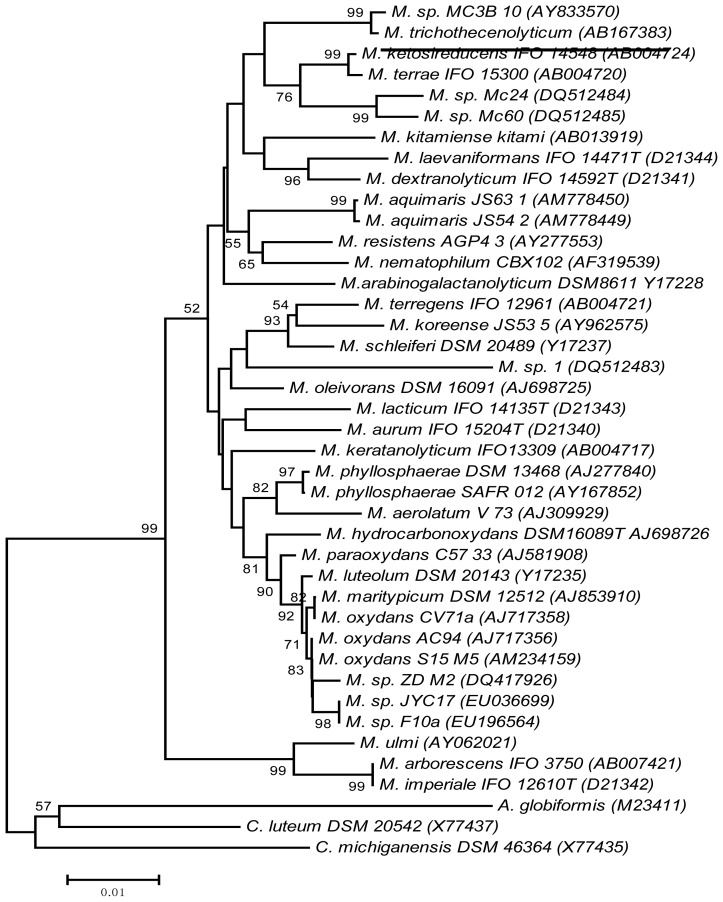
Neighbor-joining phylogenetic tree, based on *16S rDNA* gene sequences, showing the positions of strain MC3B-10 (AY833570) relative to all known *Microbacterium* species. Accession numbers of *16S rDNA* gene sequences of reference organisms are shown in parentheses. Bootstrap values (1000 replicates, >50%) are shown above the nodes. The bar indicates the relative sequence divergence (0.01 nucleotide substitutions per site). *Curtobacterium luteum* and *C. michiganense* were used as outgroups.

The reassessment of the phylogenetic position of *Microbacterium* sp. MC3B-10 based on its 16S rDNA gene, using a more robust bioinformatics approach, confirmed that its closest relative was *Microbacterium trichothecenolyticum*, with a similarity of 99.2% (underline, Figure 4). Stackebrandt and Ebers [33] established that a cutoff of 98.7% *16S rDNA* gene homology is appropriate for species differentiation within a genus. Following this criterion and given the fact that *Microbacterium* is a very tight genus with respect to *16S rDNA* gene homology between valid species [34], *Microbacterium* sp. MC3B-10 could be classified as *M. trichothecenolyticum.* However, definitive identification of this bacterial isolate requires a polyphasic approach including biochemical, physiological, chemotaxonomic, and nucleic acid-based methods, along with a range of microscopies, as previously shown for newly described *Microbacterium* species [35].

The *in-silica* analysis revealed novel strains of the genus *Microbacterium* (MC24 and MC60) that had been isolated from an intertidal environment in Brazil and were capable of synthesizing bioemulsifiers [36]. These isolates were also related to *Microbacterium* sp. MC3B-10, but at lower similarity levels (97%). It should be noted that, except for *M. resistens*, *M. hominis*, and *M. paraoxydans*, all microbacteria are considered environmental bacteria [34]. Certain *Microbacterium* species, such as *M. thalassium*, *M. halophilum,* and *M. phyllosphaerae*, occur in coastal habitats [35,37]. However, the biotechnological potential of this genus as a marine bioemulsifier producer has only been reported recently [8,36]. The occurrence of bioemulsifier-producing *Microbacterium* species in intertidal environments is not surprising, given the metabolic versatility exhibited by this genus; some of them can metabolize hydrocarbons, presumably through bioemulsifier synthesis [38,39]. In addition, the availability of hydrophobic substrates in coastal marine environments may select for bacteria capable of synthesizing surface-active molecules to enhance nutrient uptake [1].

## 3. Experimental Section

### 3.1. Production of Microbactan

*Microbacterium* sp. MC3B-10 was originally isolated from pristine rocky intertidal shores in the state of Campeche, southern Gulf of Mexico, Mexico. Microbactan was produced following a batch shake flask fermentation of an overnight culture (50 mL) of *Microbacterium* sp. MC3B-10 as previously reported [8]. The exopolymeric material was extracted from the fermented broth, redissolved in a small volume of distilled water and then dialyzed (molecular weight cutoff 12,000–14,000 Da, Spectrum^®^, CA, USA) for 72 h. The resulting material was lyophilized and kept in the dark before analysis.

### 3.2. Lipid Analysis and Fatty Acid Composition

Lipids were determined using Fourier-transform infrared spectroscopy (FT-IR) [8] and spectrophotometry, using triolein as standard lipid [40]. Fatty acid composition was investigated as follows. After mild acid hydrolysis of the exopolymer (0.7064 g) at 80 °C for 30 min, the hydrolysate was extracted with CHCl_3_:CH_3_OH:H_2_O (3:2:1) and the aqueous phase extracted two more times with CHCl_3_ (1:1). The CHCl_3_ fractions were combined and evaporated under reduced pressure [41]. Before GC-MS analysis, the sample was pre-treated as described previously [42]. The methylation of fatty acids was carried out with 5 mL of HCl-methanol at 80 °C for 30 min. The fatty acid methyl ethers were extracted with hexane and subjected to analysis. GC-MS was performed using helium as carrier gas on a Perkin Elmer Clarus 580 GC equipped with a Clarus SQ 8S mass spectrometer, equipped with an Elite-5 capillary column (30 m × 0.32 mm i.d., 0.25 μm film thickness).

### 3.3. High Performance Size Exclusion Chromatography

The molecular weight of microbactan was determined using an HPLC system Prominence Shimadzu™, a PL aquagel-OH mixed, 8 μm (Varian) guard column (U 7.5 mm × L50 mm), and a PL aquagel-OH mixed (Varian, Palo Alto, CA, USA) separation column (U 7.5 × 300 mm, operating range 10^2^–10^7^ g/mol). Elution was performed at 1 mL/min with 0.1 M ammonium acetate containing 0.03% NaN_3_, and the eluate filtered through a 0.1 μm membrane (Durapore Membrane, PVDF, Hydrophilic type VVLP, Millipore^®^, Saint Quentin en Yvelines, France). A differential refractive index (RI) detector (L2490, VWR Hitachi, Fontenay sous bois, France) and a multi-angle light scattering detector (Dawn Heleos™, Wyatt, Toulouse, France) were coupled on-line and data computed with Astra software for absolute molar mass determination.

### 3.4. Ionic Charge

The ionic charge of microbactan was assessed using a modified double diffusion technique [43]. Briefly, two 12 mm-diameter plugs were taken from Petri dishes containing 1% agar. One of the resulting wells was filled with 200 μL of microbactan solution at 1 mg/mL and the other with commercial anionic (sodium dodecyl sulphate SDS and Teepol^®^ 610S Sigma-Aldrich Química, S. de R.L. de C.V., Toluca, Mexico State, Mexico) or cationic (barium chloride and cetyltrimethylammonium bromide) surfactants supplied by Sigma-Aldrich. These surfactants were used at 20 mmol, except for barium chloride (50 mmol). The appearance of precipitation lines between the wells was indicative of the ionic character of microbactan. Plates were incubated at 28 °C and monitored daily.

### 3.5. Emulsifying Activity

The emulsifying activity of microbactan was assessed against aromatic hydrocarbons (benzene and xylene), vegetable oils (corn, olive and sunflower) and mineral, motor and crude oils, all at 1%. Oils were acquired from local commercial suppliers and the hydrocarbons were of analytical grade (J.T. Baker, Center Valley, PA, USA; E.M. Sciences, Hatfield, PA, USA). The emulsifying activity was measured by combining equal volumes of microbactan solution (1% *w*/*v*) and the target hydrophobic substrates in 12-mm-diameter glass tubes, as previously described [8]. Controls included Triton^®^ X-100 and Tween^®^ 80 (Research Organics, Cleveland, OH, USA), a plant-derived polysaccharide (gum arabic from Spectrum^®^) and xanthan gum, a bacterial polysaccharide (ICN Biomedicals, Inc, Irvine, CA, USA). All evaluations were performed in triplicate.

### 3.6. Effects of Temperature, Salinity and pH on Emulsifying Activity

The effect of temperature, NaCl content and pH on emulsifying activity of microbactan was tested using motor oil because this substrate yielded the highest levels of emulsification (~96%). The influence of temperature was determined by pretreating at desired temperatures (5, 50 and 100 °C) microbactan solutions that were either heated for 15 min in a water bath or cooled for 10 min in a freezer, before being assayed [23]. The effect of NaCl was investigated at three concentrations (3.5, 5 and 10% *w*/*v*) and pH values of 4, 7 and 10 were studied. Emulsification with motor oil was carried out at room temperature as in Item 3.5 [22,24].

### 3.7. Toxicity Test

Brine shrimp eggs (Salt Creek™, Salt Lake City, UT, USA) were hatched at 27 °C under continuous aeration and illumination in seawater prepared with sea salts (Coralife^®^, Rhinelander, WI, USA) at 38 g/L and supplemented with 6 mg/L of dried yeast [44]. Solutions of Triton^®^ X-100 and Tween^®^ 80 (Research Organics, Cleveland, OH, USA) and microbactan were dissolved in seawater at concentrations of 1000, 500, 100, 50 and 10 μg/mL [45]. The 50% Lethal Concentration (LC_50_) was determined by counting the dead nauplii after an incubation period of 24 h. Data were analyzed with the Finney computer program as described previously [27].

### 3.8. Statistical Analysis

All the experiments were run in triplicate. For the emulsification assays, a two-way analysis of variance (ANOVA) was used to assess the effect of biopolymer and controls on the hydrophobic substrates. When there was a significant difference (*p* ≤ 0.05) between microemulsion and controls, we applied the Tukey test aposteriori. These tests were performed with Sigma Stat software version 2.0 (1997).

### 3.9. Phylogenetic Reassessment

The purpose of this analysis was twofold: first, to corroborate the identification of the species of *Microbacterium* MC3B-10 based on its 16S rDNA gene (AY833570) using a more robust bioinformatics approach (see details below) with the sequences reported in [8], and second to prove if recently reported species of *Microbacterium* displaying bioemulsifying activity or originally isolated from polluted environments were phylogenetically close to *Microbacterium* sp. MC3B-10. The following additional sequences were obtained from the GenBank database, *Microbacterium* sp. Mc1 DQ512483, Mc24 DQ512484, Mc60 DQ512485, *M. aquimaris* JS54-2 AM778449, *M. aquimaris* JS63-1 AM778450, *Microbacterium* sp. F10a EU196564, *M. oleivorans* DSM 16091 AJ698726 and *M*. sp. ZD-M2 DQ417926. In total, 41 sequences (1418 bp) were aligned using the CLUSTALW algorithm implemented in MEGA 4.3 software [46]. Phylogenetic analyses were performed using the Neighbor Joining (NJ) algorithm: phylogenetic analysis using parsimony V4.0 beta 10, 2002. For NJ analyses, the evolutionary model selected was the GTR + I + G (General Time Reversible Model + Invariable sites + Gamma distribution) inferred from the program Modeltest V.3.06 [47]. Support for nodes of the NJ tree was determined by calculating bootstrap proportion values [48] based on 1000 resamplings of neighbor-joining searches. The 16S rDNA sequences of *Curtobacterium luteum* and *C. michiganense* were used as outgroup.

## 4. Conclusions

This study demonstrated that the novel *Microbacterium* sp. MC3B-10, probably indigenous to the marine intertidal zone, synthesizes an environmentally-friendly exopolymeric non-ionic glycolipoprotein capable of emulsifying aromatic hydrocarbons and oils. The functional stability of this bioemulsifier was retained for long periods of time and at a range of temperature, NaCl concentration and pH. The chemistry, activity and stability of microbactan make it useful for environmental and personal care applications. Overall, our results suggest that bioemulsifier-producing bacteria warrant intensified bioprospection in the intertidal zones. Additionally, this study corroborates the still untapped resource represented by marine microorganisms for new biosurfactants, bioemulsifiers and biopolymers. This work thus strengthens the notion that marine microbial diversity is a non-exhausted source of novel biomolecules and particularly emphasizes the biotechnological significance of *Microbacterium* species derived from the intertidal environment.

## Figures and Tables

**Table 1:** Emulsifying activity of microbactan, commercial synthetic surfactants and natural biopolymer emulsifiers on various hydrophobic substrates after 24 h of evaluation.

Hydrophobic substrate	Microbactan a	Synthetic surfactants a		Biopolymers a	
	
		Tween 80	Triton-X-100	Gum arabic	Xanthan gum
Benzene	76.9 ± 2.4	98.7 ± 0.6	54.3 ± 2.0	80.7 ± 3.0	64.7 ± 1.3
Xylene	81.5 ± 3.4	94.7 ± 6.1	98.2 ± 0.4	72.6 ± 3.4	76.8 ± 1.7
Crude oil	76.5 ± 2.1	82.9 ± 0.6	100	54.7 ± 3.7	95.0 ± 0.9
Motor oil	96.3 ± 0.1	54.9 ± 1.4	68.5 ± 6	95.2 ± 1.1	89.5 ± 3.3
Sunflower oil	84.4 ± 5.4	100	100	77.8 ± 1.5	62.4 ± 2.4
Corn oil	81.1 ± 1.6	100	100	0	90.6 ± 1.9
Olive oil	76.1 ± 0.3	100	100	96.6 ± 0.1	76.6 ± 5.4
Mineral oil	0	95.9 ± 5.6	100	60.3 ± 2.8	86.5 ± 3.8

a Results are expressed as percentages of the total height occupied by the emulsion; values are means of at least three determinations.

**Table 2:** Emulsifying activity of microbactan, commercial synthetic surfactants and natural biopolymer emulsifiers on various hydrophobic substrates after 96 h of evaluation.

Hydrophobic substrate	Microbactan a	Synthetic surfactants a		Biopolymers a	
	
		Tween 80	Triton-X-100	Gum arabic	Xanthan gum
Benzene	75.3 ± 3.8	93.2 ± 5	54.3 ± 2.1	78.9 ± 0	61.7 ± 1.4
Xylene	75.4 ± 0.5	94.1 ± 5	97.9 ± 0.5	63.7 ± 3	75 ± 1.6
Crude oil	72 ± 4.8	85.5 ± 5	100 ± 0	53.2 ± 2.4	93.8 ± 2.5
Motor oil	96.3 ± 0.1	53.5 ± 2.3	59 ± 4.2	92.8 ± 1.8	83.7 ± 3.2
Sunflower oil	83.8 ± 3.4	100	100	77.8 ± 2.1	62.4 ± 2.4
Corn oil	81.1 ± 1.6	100	100	0	91.5 ± 0.8
Olive oil	76.1 ± 0.5	100	100	96.6 ± 0.6	78.2 ± 2.6
Mineral oil	0	91.2 ± 2.3	100 ± 0	56.8 ± 2.3	82.9 ± 2

a Results are expressed as percentages of the total height occupied by the emulsion; values are means of at least three determinations.

**Table 3:** Anti-crustacean activity of surfactants against *Artemia salina* nauplii.

Surfactant	LC_50_ (μg/mL)
Microbactan	>1000
Triton X-100	100.3 ± 3.8
Tween 80	>1000
